# The SPO11-C631T gene polymorphism and male infertility risk: a meta-analysis

**DOI:** 10.1080/0886022X.2016.1274661

**Published:** 2017-01-04

**Authors:** Zheng-Ju Ren, Peng-Wei Ren, Bo Yang, Jian Liao, Sheng-Zhuo Liu, Kun Fang, Shang-Qing Ren, Liang-Ren Liu, Qiang Dong

**Affiliations:** a Department of Urology, Institute of Urology, West China Hospital, Sichuan University, Chengdu, Sichuan, China;; b Department of Evidence-Based Medicine and Clinical Epidemiology, West China Hospital, Sichuan University, Chengdu, Sichuan, China

**Keywords:** SPO11, SPO11 gene, polymorphism, male infertility, meta-analysis

## Abstract

To evaluate the association between the SPO11 gene C631T polymorphism and the risk of male infertility. We conducted a search on PubMed, Embase, Web of Science, Chinese National Knowledge Infrastructure (CNKI), China biology medical literature database (CBM), VIP, and Chinese literature database (Wan Fang) on 31 March 2016. Odds ratio (OR) and 95% confidence interval (95%CI) were used to assess the strength of associations. A total of five studies including 542 cases and 510 controls were involved in this meta-analysis. The pooled results indicated that the SPO11 gene C631T polymorphism was significantly associated with increased risk of male infertility (TT + CT vs. CC: OR = 4.14, 95%CI = 2.48–6.89; CT vs. CC: OR = 4.34, 95%CI = 2.56–7.34; T vs. C: OR = 4.35, 95%CI = 2.58–7.34). Subgroup analysis of different countries proved the relationship between SPO11 gene C631T polymorphism and male infertility risk in Chinese, but not in Iranian peoples. In conclusion, this study suggested that SPO11 gene C631T polymorphism may contribute as a genetic factor susceptible to cause male infertility. Furthermore, more large sample and representative population-based cases and well-matched controls are needed to validate our results.

## Introduction

Infertility is defined as the failure of a couple to achieve pregnancy after one year of unprotected regular sexual intercourse, which affects approximately 15% of all couples trying to conceive a child.^1^^,^^2^ In addition to environmental and lifestyle risk factors, genetic causes such as chromosomal aberrations and single-gene mutations also play an important role in male infertility.^3–6^ Studies have shown that genetic abnormalities account for approximately 15% of male infertility.^7^^,^^8^ Several studies suggest the SPO11 gene as a candidate gene for male infertility.

The Spo11 gene is localized on chromosome 20q13.2–q13.3 and Spo11 is an evolutionary conserved protein, involved in the formation of DNA double strand breaks (DSB), which can initiate meiotic recombination and promote pairing and synaptonemal complex (SC) formation between homologous chromosomes.^9–11^ When SPO11 expression and DSB number are reduced below heterozygosity levels, the chromosome synapsis is delayed, chromosome tangles form and defective cells are removed by apoptosis at epithelial stage IV.^12^ Meiosis is an essential and complex process in gametogenesis and SPO11 gene is one of the important genes, which is involved in meiosis. The disruption of SPO11 gene in mouse by homologous recombination leads to severe gonadal abnormalities from defective meiosis and apoptotic death during early prophase, resulting in male infertility.^13^^,^^14^ Several studies have studied the association between SPO11 C631T gene polymorphism and male infertility. However, the majority of these had small patient sample sizes, and the results remain inconclusive rather than consistent. Therefore, to evaluate the relationship between the SPO11 C631T gene polymorphism and risk of male infertility, we have conducted a meta-analysis from all relevant scientific literatures.

## Materials and methods

### Searching strategy

**Figure 1.**
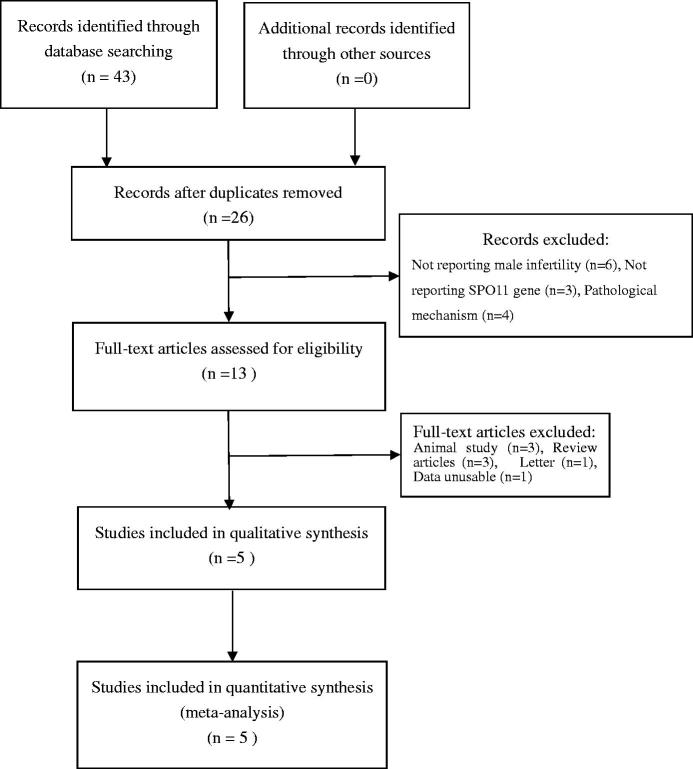
Flowchart showing the study selection.

Two authors independently conducted a systematic literature search, using PubMed, Embase, Web of Science, Chinese National Knowledge Infrastructure (CNKI), China biology medical literature database (CBM), VIP, and Chinese literature database (Wan Fang) up to 31 March 2016. Search terms were as follows: “SPO11 gene”, “polymorphism, mutation, or variant”, “male infertility”. In addition, the references of retrieved articles were reviewed to identify other eligible studies missed by the search. The search was limited to human subjects. The search strategy flowchart is shown in Figure 1.

### Inclusion criteria

Only for the study meeting the following inclusive selection criteria were eligible (1) studies with full text articles; (2) case–control studies evaluating the association between SPO11 gene polymorphism and susceptibility to male infertility; (3) the genotype distributions were available for both cases and controls; (4) no overlapping data. For the studies with the same or overlapping data by the same authors, we selected the ones with the most subjects. (5) The published language was English or Chinese.

### Exclusion criteria

Studies were excluded if any of the following criteria existed: (1) not for the association between SPO11 gene polymorphism and the male infertility risks; (2) studies with partial unusable data; (3) animal studies, review articles, meta-analyses, conference abstracts, or editorial articles.

### Quality assessment

The Newcastle-Ottawa Scale (NOS)^15^ was used to assess the quality of the included studies. The NOS contains eight items for both cohort and case–control studies. It is categorized into three aspects including selection, comparability, and exposure for case-control studies. A ‘‘star’’ rating system is used to judge the methodological quality. Selection has a maximum of four stars, Comparability has a maximum of two stars, and Exposure has a maximum of three stars. Scores ranged from zero stars (worst) to nine stars (best), the quality of each study was graded as low (0–3), moderate (4–6), and high (7–9). Discrepant opinions were resolved by discussion and consensus.

### Data extraction strategy

Two authors extracted the relevant data independently complying with the inclusion criteria. Extracted data were entered into a collection form and checked by a third author. Disagreement was solved by discussion and consensus finding. In the present meta-analysis, we collected the following variables for each study: (1) the first author’s name, year of publication, region, genotyping method; (2) sample size of the study case and control groups; (3) the results of the Hardy–Weinberg equilibrium test.

### Statistical analysis

The strength of the relationships between SPO11 gene polymorphism and the male infertility risks were assessed using OR and corresponding 95%CI. The pooled ORs were performed for allele comparison model, dominant model and codominant model, respectively. Heterogeneity assumption was tested using the Chi-square-based *Q* test. The heterogeneity was considered significant when *p* < .10, and *I*^2^ values of 25%, 50%, and 75% corresponded to low, medium, and high levels of heterogeneity. A fixed-effect model was applied to evaluate the summary OR, when the *p* values for heterogeneity was >.10 and *I*^2 ^<^ ^50%. In contrast, we applied the random-effect model if *p* ≤ .10 or *I*^2 ^≥^ ^50%. The significance of the pooled OR was determined by the *Z*-test, and *p* < .05 was considered as statistically significant. The statistical analysis was performed with Reviewer Manager 5.3 and Stata 12.0. The potential publication bias was estimated using Begg’s test, Egger’s test, and funnel plots. Sensitivity analysis was performed to evaluate the stability of the results. The pooled ORs were estimated by excluding one study each time to evaluate the influence of single study.

## Results

### Study inclusion and characteristics

A total of 43 results were retrieved after first search in PubMed, Embase, Web of Science, Chinese National Knowledge Infrastructure (CNKI), China biology medical literature database (CBM), VIP, and Chinese literature database (Wan Fang). Of these studies, after the first screening, 38 studies were excluded based on inclusion and exclusion criteria. Finally, after our careful selection, five case–control studies considering 542 cases and 510 controls were included in this meta-analysis.^16–20^ In particular, there were three studies conducted in Chinese and two in Iranian populations. The years of publication ranged from 2011 to 2015. The Hardy–Weinberg test (HWE) was performed on all of the included studies, and the results showed that the SPO11 gene genotype frequencies of five studies were in HWE in the controls. The detailed characteristics of all the included studies are shown in Table 1. And, the quality of studies based on the NOS score is presented in Table 2.

**Table 1.** Main characteristics of studies included in the meta-analysis.

			Genotyping	Genotype (CC/CT/TT)		
Authors	Year	Ethnicity	Method	Infertility	Control	HWE(*p*)
Zhang et al^18^	2011	Chinese	PCR-RFLP	65/8/0	114/3/0	.888
Karimian et al^16^	2015	Iranian	PCR-RFLP	100/0/0	99/1/0	.960
Ghalkhani et al^17^	2014	Iranian	PCR-RFLP	100/12/1	49/1/0	.943
Han et al^20^	2012	Chinese	PCR-RFLP	34/6/0	44/1/0	.940
Feng et al^19^	2015	Chinese	PCR	189/27/0	195/3/0	.914

**Table 2.** Quality assessment for all the included studies.

First authors	Publishing year	Selection	Comparability	Exposure	Total
Zhang	2015	★★★	★	★★	6
Karimian	2015	★★★	★	★★	6
Ghalkhani	2014	★★	★	★★	5
Han	2013	★★★	★	★★	6
Feng	2012	★★★	★	★★	6

### Meta-analysis results

**Figure 2.**
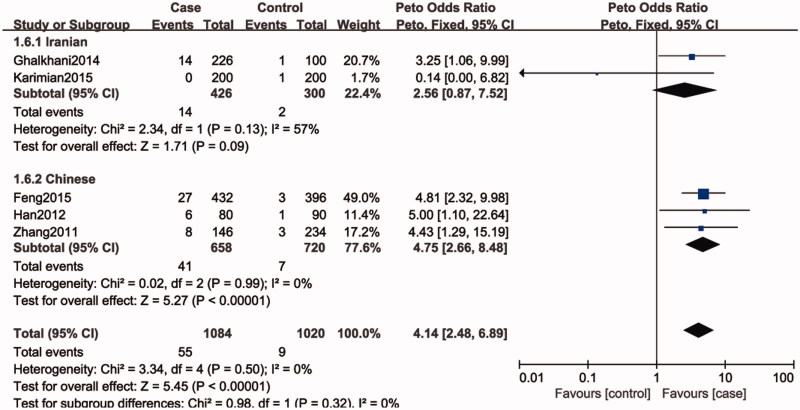
Forest plot of the studies assessing the association between SPO11 C631T gene polymorphisms and male infertility (subgroup analyses for the Iranian and Chinese: allele model: T vs. C).

**Figure 3.**
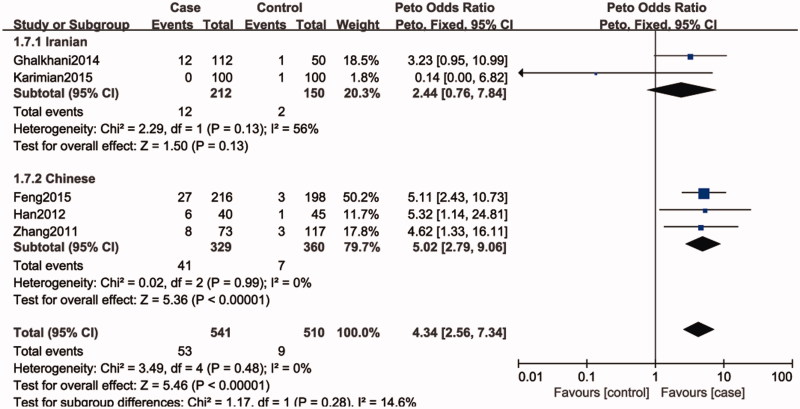
Forest plot of the studies assessing the association between SPO11 C631T gene polymorphisms and male infertility (subgroup analyses for the Iranian and Chinese: codominant model: CT vs. CC).

**Figure 4.**
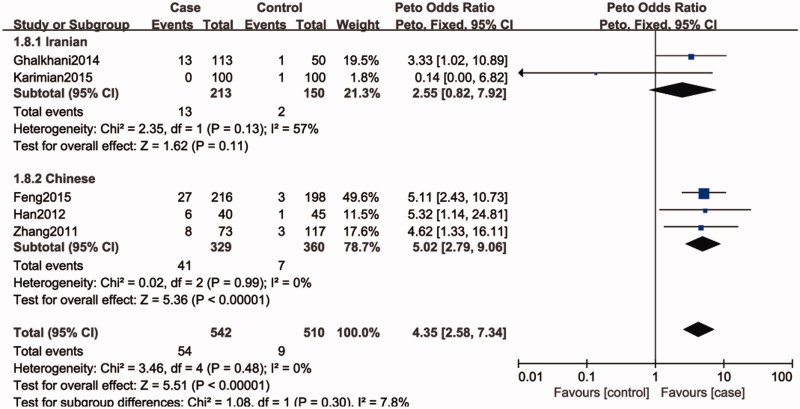
Forest plot of the studies assessing the association between SPO11 C631T gene polymorphisms and male infertility (subgroup analyses for the Iranian and Chinese: dominant model: TT + CT vs. CC).

Five studies included 1052 individuals, which totally evaluated the influence of SPO11 C631T polymorphism on the risk of male infertility. Among the individuals included, only one subjects had TT genotype in C631T location. Therefore, we could only compare TC and TT-aggregated genotypes vs. CC, CT genotype vs. CC genotypes and T allele vs. C allele. There was no significant heterogeneity in any genotype contrasts among the studies, and fixed-effects models were applied. Overall, the results revealed a significant association between the SPO11 C631T polymorphism and male infertility risks (TT + CT vs. CC: OR = 4.14, 95%CI = 2.48–6.89; CT vs. CC: OR = 4.34, 95%CI = 2.56–7.34; T vs. C: OR = 4.35, 95%CI = 2.58–7.34) (Figures 2–4).

In a subgroup analysis by ethnicity, we observed a significant association between SPO11 C631T polymorphism and male infertility risks in Chinese (TT + CT vs. CC: OR = 5.02, 95%CI = 2.79–9.06; CT vs. CC: OR = 5.02, 95%CI = 2.79–9.06; T vs. C: OR = 4.75, 95%CI = 2.66–8.48), whereas there was no significantly elevated infertility risks associated with the SPO11 C631T polymorphism and male infertility in Iranian population (TT + CT vs. CC: OR = 2.55, 95%CI = 0.82–7.92; CT vs. CC: OR = 2.44, 95%CI = 0.76–7.84; T vs. C: OR = 2.56, 95%CI = 0.87–7.52).

### Sensitivity analyses and publication bias

**Figure 5.**
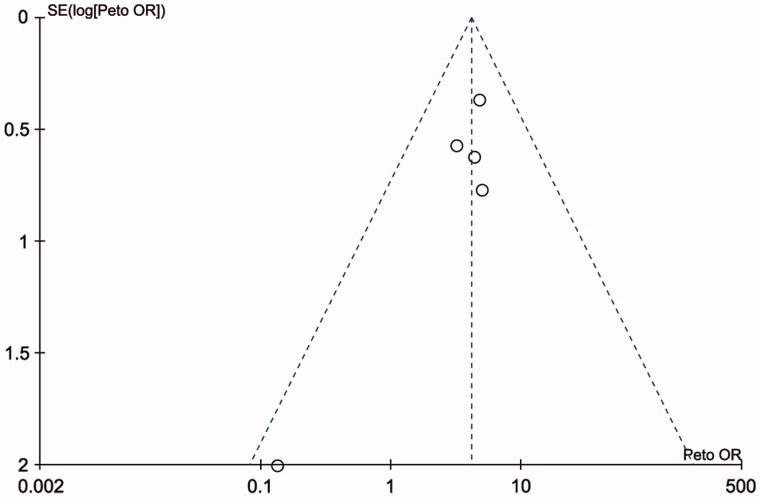
Funnel plot of the studies assessing the association between SPO11 C631Tgene polymorphisms and male infertility (allele model: T vs. C).

**Figure 6.**
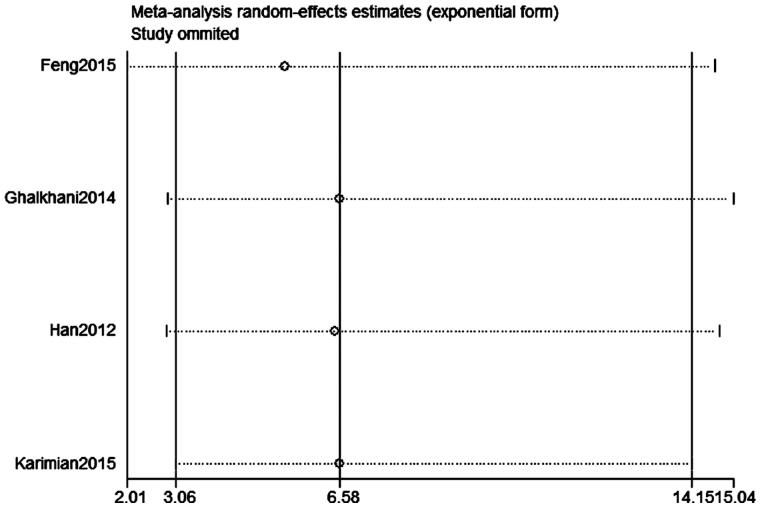
Sensitivity analysis diagram for each study used to assess the relative risk estimates for the SPO11 C631T gene polymorphism and male infertility in all the included studies (allelic model: T allele vs. C allele).

**Figure 7.**
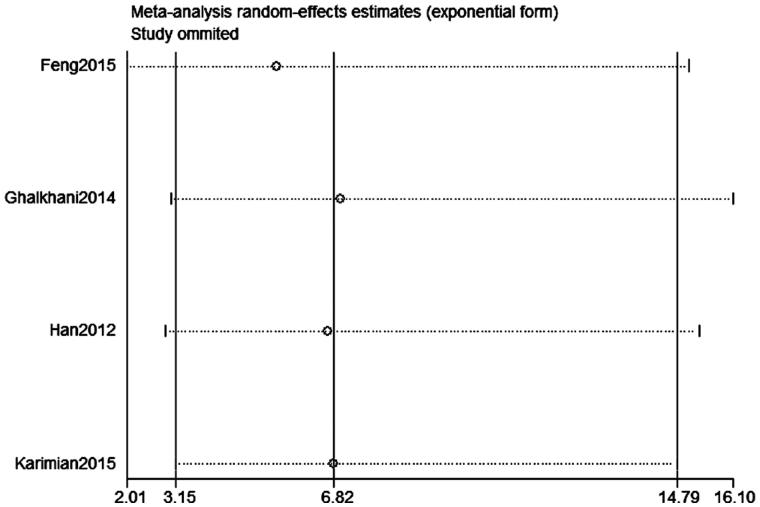
Sensitivity analysis diagram for each study used to assess the relative risk estimates for the SPO11 C631T gene polymorphism and male infertility in all the included studies (codominant model: CT vs. CC).

Publication bias was assessed on SPO11 C631T polymorphism by Begg’s test, Egger’s test, and funnel plots, the shape of the funnel plot did not indicate any evidence of obvious asymmetry in the allelic contrast models on the SPO11 C631T polymorphism (Figure 5). In addition, Egger’s linear regression analysis also suggested that there was no evidence of publication bias (*p* = .506 for an allelic contrast model, *p* = .529 for a codominant model, *p* = .491 for a dominant model, respectively) (Table 3). The sensitivity analyses were conducted to calculate the pooled ORs through omitting one study each time, and the results showed no individual study influenced the overall pooled ORs (Figures 6,7). That is to say, the results of this meta-analysis are relatively stable.

**Table 3.** Publication bias test for SPO11 C631T polymorphism.

	Egger’s test			Begg’s test
Comparisons	Coefficient	*p* Values	95%CI	*p* Values
T vs. C	0.006	0.841	−0.104 to 0.116	.734
CT vs. CC	0.010	0.820	−0.157 to 0.177	1.000
CT + TT vs. CC	0.008	0.851	−0.160 to 0.176	1.000

## Discussion

This meta-analysis was performed to provide a clear understanding of the SPO11 C631T gene polymorphisms and risk of male infertility. Our results of this meta-analysis suggest that genetic variations of SPO11 C631T may contribute to susceptibility to male infertility.

In the present study, the overall results showed that SPO11 gene polymorphism could increase the risk of male infertility (TT + CT vs. CC: OR = 4.14, 95%CI = 2.48–6.89; CT vs. CC: OR = 4.34, 95%CI = 2.56–7.34; T vs. C: OR = 4.35, 95%CI = 2.58–7.34). It reveals that individuals with the variant T allele may have a higher risk for male infertility than those carrying C homozygote. In our study, there is no evidence of heterogeneity across studies, even though we included populations of China and Iran. Ethnicity was not associated with the SPO11 C631T allele frequency, making it unlikely that ethnicity was a confounder in this meta-analysis. Nevertheless, in the subgroup analysis of ethnicity, we found that SPO11 C631T had an effect on increase in the male infertility risk in Chinese, while the susceptibility to male infertility was not observed in Iranian population. This may be due to small sample size in the studies and different genetic backgrounds in Iranian population.

SPO11 is accepted as a meiosis-specific protein which could generate double-strand breaks on chromosomal DNA and initiates meiotic recombination in a wide variety of organisms. The expression of SPO11 gene increases during meiosis.^21^ It has been shown that in rats, SPO11 knockout activates meiotic arrest in zygotene spermatocytes, resulting the disruption of spermatogenesis and normal testis development.^13^^,^^14^ Therefore, any change or mutation in SPO11 gene may cause infertility in men. Karimian et al. have studied SPO11 gene polymorphism in 100 infertile and100 fertile men in Iran. Results showed that only one of the fertile subjects had CT genotype in C631T location and the rest of the subjects (100 infertile and 99 fertile men) had CC genotype. Conversely, Ghalkhani et al. performed another study in Iranian population. They reported that C361T was significantly associated with male infertility in all populations except oligozoospermic cases from the Center region in Iran. Our present study revealed that SPO11 gene polymorphism significantly associated with male infertility risk, which is in good agreement with previous observations on Chinese population samples. The inconsistency between the studies could arise from race, geography or genetic differences of the study population. However, we enrolled only five studies in the present study. Well designed, unbiased, and large case-control studies should be performed to acquire a more precise association between the SPO11 gene polymorphism and male infertility risk.

When interpreting the results of the current study, some limitations should be taken with cause. First, only five studies were included in the meta-analysis, the sample size of included published articles was small, and so sufficient data was unavailable. Second, we did not estimate the potential interactions among gene–gene, gene–environment, due to the lack of information in the original studies. Third, other clinical data such as source of control, age, semen quality, and so on, were not analyzed also due to the lack of information. Finally, some inevitable publication bias might exist in the results because only published studies were retrieved although the funnel plot and Egger’s test indicated no remarkable publication bias.

## Conclusions

In summary, this meta-analysis provides evidence that the SPO11 C631T polymorphisms may contribute to genetic susceptibility to male infertility risk. Nevertheless, large-scale, well-designed, and population-based studies are still needed to investigate the findings.
